# Application analysis of Six Sigma DMAIC model in new nurse training and effects of nutritional diet nursing on postoperative nutritional status in the elderly patients with fracture

**DOI:** 10.3389/fmed.2025.1657619

**Published:** 2025-09-23

**Authors:** Fangfang Xiong, Jiao Tang, Han Han, Xue Wang, Jing Cao, Xianglei Wang, Qinghua Zhao

**Affiliations:** ^1^Department of Nursing, The First Affiliated Hospital of Chongqing Medical University, Chongqing, China; ^2^School of Nursing, Chongqing Medical University, Chongqing, China; ^3^Department of Orthopedics, The First Affiliated Hospital of Chongqing Medical University, Chongqing, China; ^4^Department of Oncology, The First Affiliated Hospital of Chongqing Medical University, Chongqing, China; ^5^Department of Nephrology, The First Affiliated Hospital of Chongqing Medical University, Chongqing, China

**Keywords:** DMAIC, fracture, nurse training, nutritional diet nursing, quality management, Six Sigma

## Abstract

**Background:**

In the current medical system, the insufficient efficiency of the standardized training for new nurses is a major issue. At the same time, elderly patients with fractures are prone to nutritional deficiencies after surgery, which seriously affects their rehabilitation process and treatment outcomes.

**Objectives:**

This study evaluated the application of the Six Sigma DMAIC model in the training of new nurses, as well as the impact of nutritional diet nursing on the postoperative nutritional status in the elderly patients with fractures.

**Methods:**

This was a prospective intervention study. From September 2021 to September 2022, 260 newly recruited female nurses were randomly divided into the study group and the control group using a random number table method. The control group received traditional training management model, while the study group received training using the Six Sigma DMAIC model. In addition, from November 2022 to November 2023, a total of 80 elderly patients with fractures received treatment were divided into two groups: one group received conventional nursing, and the other group received nutritional diet nursing.

**Results:**

Two years later, the study group outperformed the control group in terms of theoretical and practical assessment scores, critical thinking ability, self-efficacy, and training satisfaction, with significant difference (*p* < 0.05). Compared to the conventional nursing group, the nutritional diet nursing group had shorter incision drying time, shorter drainage placement time, and shorter healing time, with significant difference (*p* < 0.01). After nursing, the levels of albumin, prealbumin, total protein, and immunoglobulins were higher in the intervention group compared to the routine nursing group, with significant difference (*p* < 0.05). Compared to the routine nursing group, the intervention group also had a lower incidence of complications, with significant difference (*p* < 0.05 and *p* = 0.03).

**Conclusion:**

The Six Sigma DMAIC model effectively improves the quality of nurse training, while nutritional diet nursing enhances postoperative healing, nutritional status, and immune function in elderly fracture patients.

## Introduction

The newly recruited nurses are the new members of the nursing team. Through training, they acquire the basic theories, knowledge, and skills necessary for clinical nursing work and possess good professional ethics and comprehensive nursing capabilities required for providing high-quality nursing services. This is the current focus of clinical nursing management (1). At the same time, with the improvement of social living standards, the public’s requirements for medical and nursing services are also constantly increasing (2). Nurses trained by traditional management and training methods no longer meet the needs of society. The medical industry urgently needs to introduce new nursing management and training models (3).

Six Sigma is a quality management strategy aimed at enhancing the quality of processes and focusing on identifying and eliminating defects (4). Defects are regarded as any factor that causes dissatisfaction, including unnecessary processes and services (5). It employs a structured problem-solving approach consisting of the five words “define, measure, analyze, improve, and control” (6).

From the literature research, it can be seen that the success of the Six Sigma DMAIC method is not only reflected in the manufacturing industry but also in the medical field. This method has been applied in these fields (7, 8), such as for improving emergency procedures and supporting medical services (9). However, there are very few reports on the application of the Six Sigma DMAIC model in nursing teaching.

Fracture refers to the destruction or interruption of the integrity or continuity of the bone (10). Due to the slow absorption of bone calcium and the weakening of bone quality, the incidence of fractures is higher in the elderly population, and its clinical manifestations include local pain and swelling (11). In clinical practice, surgical treatment and routine nursing interventions are usually adopted to promote the recovery of patients, but the traditional postoperative nursing interventions have deficiencies in terms of nutritional intake awareness (12).

Nutritional diet nursing can be based on the assessment of the patient’s nutritional status, referring to the patient’s protein level and blood lipid levels, and other development conditions, to formulate a reasonable individualized nutritional care intervention plan, providing corresponding nursing guidance to patients to ensure their nutritional intake (13).

This study aimed to assess the effect of the Six Sigma DMAIC model in quality management of new nurse training, as well as the impact of nutritional diet nursing on postoperative nutritional status in elderly patients with fractures.

## Materials and methods

### General data

This was a prospective intervention study. The subjects of this study were 260 nurses who were newly recruited to the hospital from September 2021 to September 2022. All the nurses were female and were randomly divided into the study group (SG) and the control group (CG), with 130 nurses in each group. The 130 nurses in the SG ranged in age from 21 to 26 years, with an average age of 22.62 ± 3.08 years, which contained 60 junior college students, 40 undergraduate students, and 30 graduate students. The 130 nurses in the CG ranged in age from 20 to 26 years, with an average age of (22.58 ± 2.87) years, which contained 62 junior college students, 39 undergraduate students, and 29 graduate students. The general data of nurses in both groups were entered into statistical software for processing, and the difference between the groups was not significant (*p* > 0.05).

In addition, 80 elderly patients with fractures treated in our hospital from November 2022 to November 2023 were selected and divided into group A (40 cases) and group B (40 cases) according to the random number table method. Group A included 20 males and 20 females, aged 61–78 years, with a mean age of (69.03 ± 8.02) years. There were 18 males and 22 females in group B, aged 60–80 years, with a mean age of (68.87 ± 7.86) years. There was no significant difference between the two groups (*p* > 0.05). The inclusion criteria are as follows: (1) X-ray confirmed fracture patients. (2) Good nutritional status before admission. (3) All patients and their families gave informed consent to this study. The exclusion criteria are as follows: (1) Accompanied by systemic infectious diseases. (2) Patients with abnormal mental function. (3) Patients with autoimmune diseases.

### Randomization and blinding

A group randomization design was adopted for random grouping. The random allocation sequence was generated by a computer. The allocation confidentiality measures were achieved through sequential numbering, sealing, and opaque envelopes. After being deemed to meet the inclusion criteria, nurses and patients were randomly assigned to each group in a 1:1 ratio. This study was single-blind, and the participants were unaware of the allocation.

### Training setup

The standardized training period for both groups of nurses was 6 months. The pre-job training lasts for 1 week, covering theoretical knowledge and skills such as laws and regulations, nursing safety, management of nursing adverse events, communication skills, and personal protection. The training outlines were the same. They were all formulated strictly in accordance with the “Trial Outline for Standardized Training of Newly Recruited Nurses” issued by the National Health Commission of China and in combination with the characteristics of the patients admitted to the hospital. The training content includes basic theoretical knowledge, basic nursing operation techniques, professional theoretical knowledge, specialized nursing operations, and the clinical practical ability of nursing staff.

### Teaching methods

The CG received the traditional training management mode. During their clinical training in the departments, the trainees were guided by the teaching staff. At the same time, the nursing department conducted a centralized theoretical lecture once a month and arranged post-class examinations. Once a week, a collective operation demonstration was held. The nursing operation training room was open 24 h a day, every week, for nurses to practice independently. Before the assessment, the teachers observed, corrected, and demonstrated the problems in the operations. If necessary, they would conduct further training. An operation assessment was conducted once a month.

The SG received training based on the Six Sigma DMAIC model. The specific methods included the following:

1. Definition (D): This management training activity was initially determined, with its main purpose being to standardize the training process for nurses and to enhance their ability to adapt to the working environment and quickly master their nursing duties. Based on factors such as the nurses’ age, experience, and educational level, targeted training plans were formulated, and the costs required for training to proficiently master the job system, professional knowledge, and skills were determined. The key points of the management training program include the cultivation of professional ethics, training of work capabilities, training of professional knowledge and skills, problem-solving abilities, and the ability to handle emergencies, as well as writing of nursing documents.
2. Measurement (M): A specialized training team for new nurses was established, led by the head nurse and consisting of teachers and key nursing staff. The original nursing quality control group of this department and the hospital’s nursing quality control organization conducted spot checks on the nursing quality of the new nurses and evaluated their comprehensive abilities.
3. Analysis (A): The new nurse management training team held regular meetings to analyze the deficiencies in the management training. Through the joint efforts of the team members, they tried to identify the causes of the problems, understand the strengths and weaknesses of each new nurse from multiple perspectives, and point out the existing deficiencies.
4. Improvement (I): Based on the analysis results of the previous stage, the improvement measures were discussed and implemented. In actual operation, the institution found that the key points of improvement mainly included the following aspects: (a) Improvement of the planning work, the planning work was optimized to ensure its predictability and science, and the design of key training projects. (b) Improvement of the human resources system, the training system for new nurses was established and perfected, and the backbone force was selected to set the benchmark for nurses and play the role of the leader. At the same time, the quality control team closely supervised the training results of nurses. (c) Improvement of the work of the management team, and the head nurse played a leading role in the work, actively communicated with the superior management, coordinated the management between departments, and ensured that the training objectives could be achieved.
5. Control (C): The main work in the control stage was to constantly optimize and adjust the structure of the nursing team, emphasize the individual job responsibilities of nursing staff, promptly correct deviations, and pay attention to the skills and responsibilities of the nurses.

### Nursing methods

Routine nursing intervention was performed in group A, including admission education, routine nursing treatment of complications, fracture surgery, cognitive level intervention, postoperative pain nursing intervention, complication nursing, and rehabilitation training.

Based on the routine nursing, patients in group B received nutritional diet nursing intervention. Nutritional diet nursing regards nutritional support as one of the core contents of nursing, emphasizing the formulation of individualized dietary plans based on the patient’s specific conditions (such as age, fracture type, surgical method, and nutritional status). Through reasonable nutritional intake, it meets the special nutritional needs of patients after surgery, promotes fracture healing, enhances immune function, and reduces the occurrence of complications. It not only focuses on short-term nutritional improvement of patients but also pays attention to the maintenance of long-term nutritional status and the optimization of rehabilitation effects.

1. Assessment of the nutritional status of the patient. The nutritionist in charge of nutrition and the nurse in charge of care jointly completed the nutritional assessment of the patient and used the patient’s protein and fat content as the reference basis.
2. Development of personalized nutritional diet programs. Based on the patient’s own condition, the nurses evaluated patients’ target energy and various nutrient requirements and nutritional metabolism and formulated a personalized nutritional diet intervention plan. The plan also increases the supply of high-quality protein (with a content of ≥50%) according to the patient’s target energy. The nutritional diet program mainly included a reasonable proportion of the three major nutrients (fat, sugar, and protein), calorie requirements, protein sources, and proportions and was adjusted according to each patient’s taste, dietary intake, and eating habits. A reference recipe was formulated. The nurse guided the patient to eat more food containing trace elements such as manganese, iron, and zinc to enhance the patient’s immune function.
3. Implementation and supervision of nutritional and dietary nursing plan. The nutritionist and the responsible nurse jointly formulated a nutrition and dietary care plan, clearly defined the postoperative dietary recommendations for the patient. They also learned and observed the specific recovery and dietary conditions of the patient from the attending nurse and adjusted the nutrition and dietary intervention plan based on the obtained information.
4. The nurses meticulously recorded the patients’ daily dietary intake, encouraged the patients to follow the nutritional diet plan on time and in the correct amount, closely monitored the patients’ detailed conditions, and promptly reported cases such as diarrhea and vomiting to the doctors for appropriate treatment.

The intervention cycle for patients in both groups was 20 days.

### Observation indicators

1. After 2 years of training, the theoretical and practical assessment scores of the two groups were compared. The former included four parts, namely, specialist theoretical knowledge, basic nursing, emergency nursing, and life nursing, with 25 points for each part, and the total score was 100. The higher the score, the better the grasp of theoretical knowledge was. The practical assessment included four parts: vital sign monitoring, intravenous infusion, wound dressing change, and drainage tube nursing. Each part was 25 points, and the total score was 100 points. The higher the score, the better the practical operation ability was.
2. California critical thinking disposition inventory 2000 (CCTDI 2000) was adopted to evaluate the critical thinking ability of nurses 2 years after training (14). The scale included seven dimensions, including truth seeking, open-mindedness, analytical reasoning, systematicness, confidence, questioning, and maturity. There were 10 items in each dimension, each item was 1–6 points, and each dimension was 10–60 points. The higher the score, the stronger the critical thinking ability of nurses was.
3. General Self-Efficacy Scale (GSES) was used to evaluate nurses’ self-efficacy 2 years after training (15), which included 10 dimensions, and was scored with 1–4 points for each dimension. The higher the score, the higher the level of self-efficacy was.
4. After training, a self-made questionnaire was used to investigate the overall training satisfaction of newly admitted nurses, which was divided into three levels: very satisfied, satisfied, and unsatisfied (very satisfied + satisfied)/number of cases in the group ×100.0% = satisfaction.
5. The postoperative wound healing conditions of the two groups were compared, including incision drying time, drainage placement time, and incision healing time.
6. Nutritional status: 2 mL of fasting blood was collected from patients in the morning before and after the intervention, centrifugation at a rate of 3,000 r/min for 10 min, serum was separated, and serum albumin (ALB), prealbumin (PA), and total protein (TP) were detected by an automatic biochemical analyzer.
7. 2 mL of fasting blood was collected from patients in the morning before and after the intervention, centrifuged at a rate of 3,000 r/min for 10 min, serum was separated, and the changes in serum IgA, IgG, and IgM levels were detected by an automatic biochemical analyzer.
8. The occurrence of complications such as incision skin cracking, incision skin infection, and incision skin necrosis was compared between the two groups.

### Statistical analysis

Data were input into SPSS 18.0 statistical software for data processing, and measurement data were exhibited as mean ± standard deviation, compared with *t*-test. The count data were expressed as a rate (%), and the *χ*^2^ test was used for comparison between groups. A *p*-value of <0.05 was considered statistically significant.

## Results

### Theoretical and practical assessment scores of the two groups

**Figure 1:**
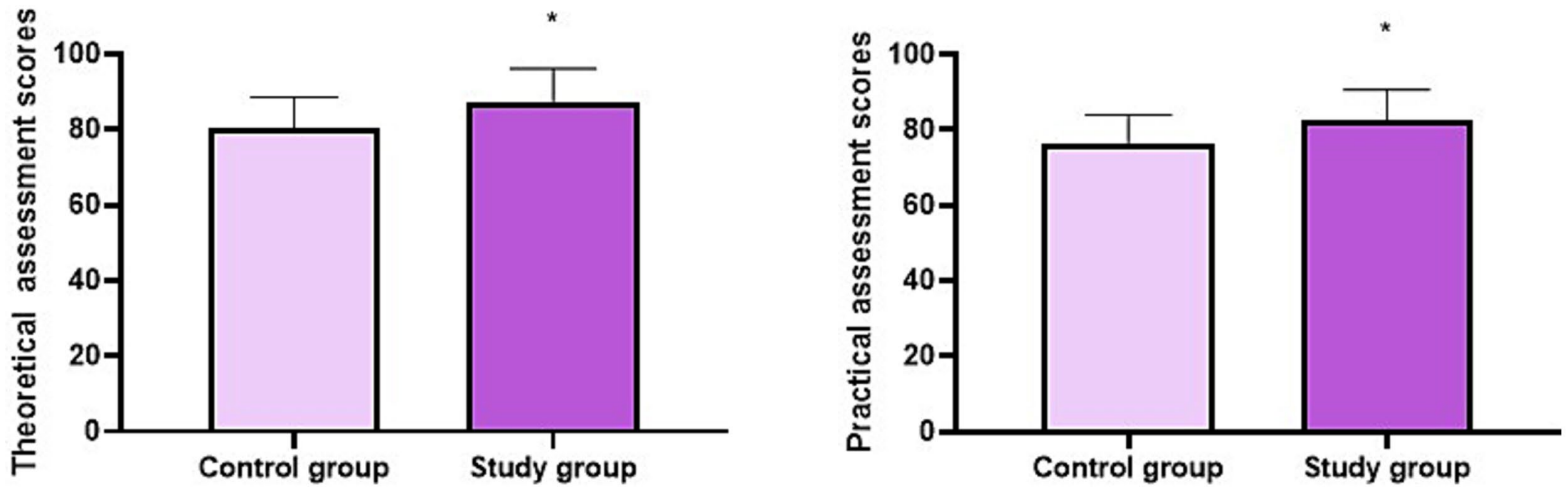
Theoretical and practical assessment scores of the two groups. ^*^p < 0.05.

After 2 years of training, the theoretical and practical assessment scores of the SG were higher compared to the CG (*p* < 0.05, Figure 1).

### CCTDI 2000 scores of the two groups

**Figure 2:**
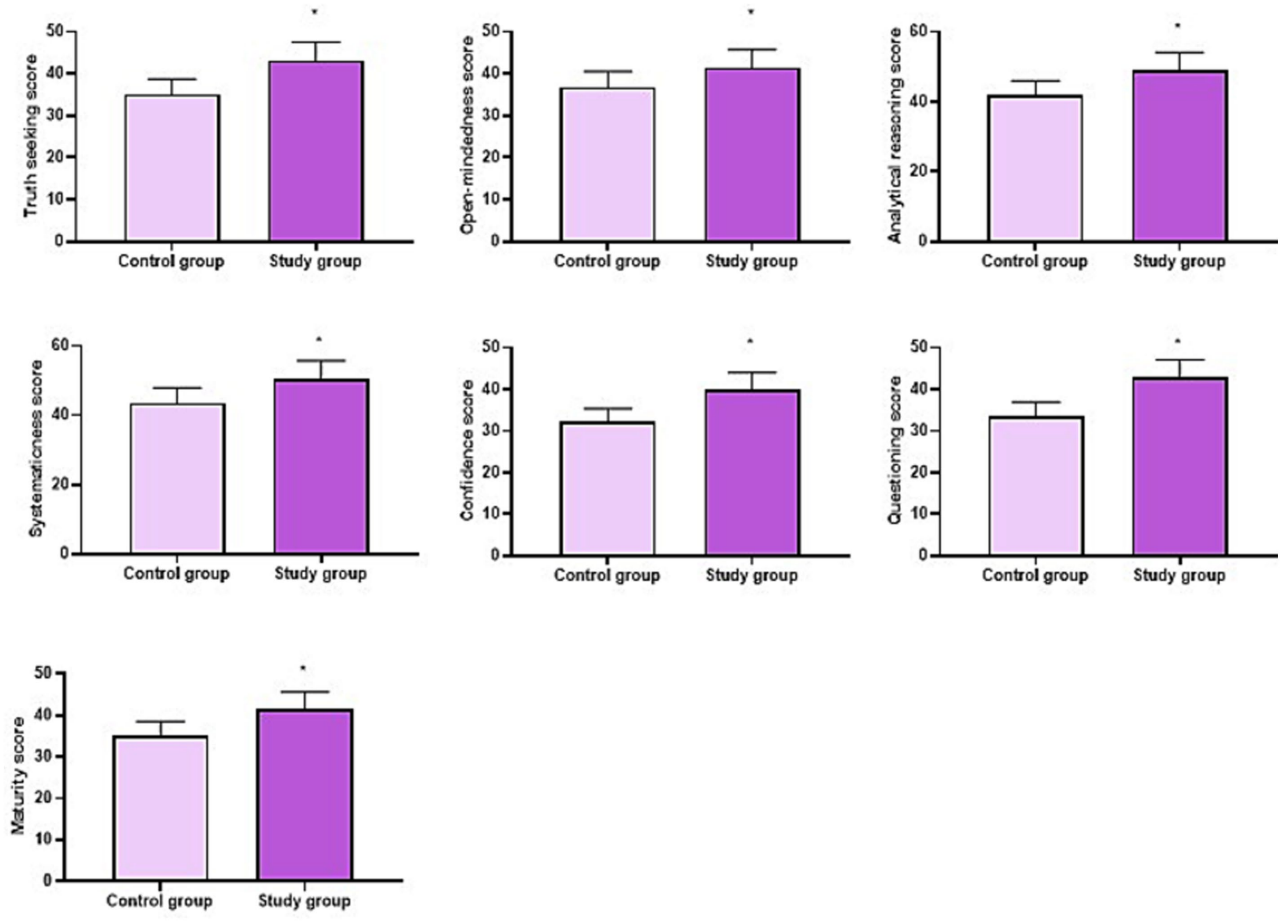
CCTDI 2000 scores of the two groups. ^*^p < 0.05.

After 2 years of training, CCTDI 2000 scores of all dimensions in the OG were higher relative to the CG (*p* < 0.05, Figure 2).

### GSES score of the two groups

**Figure 3:**
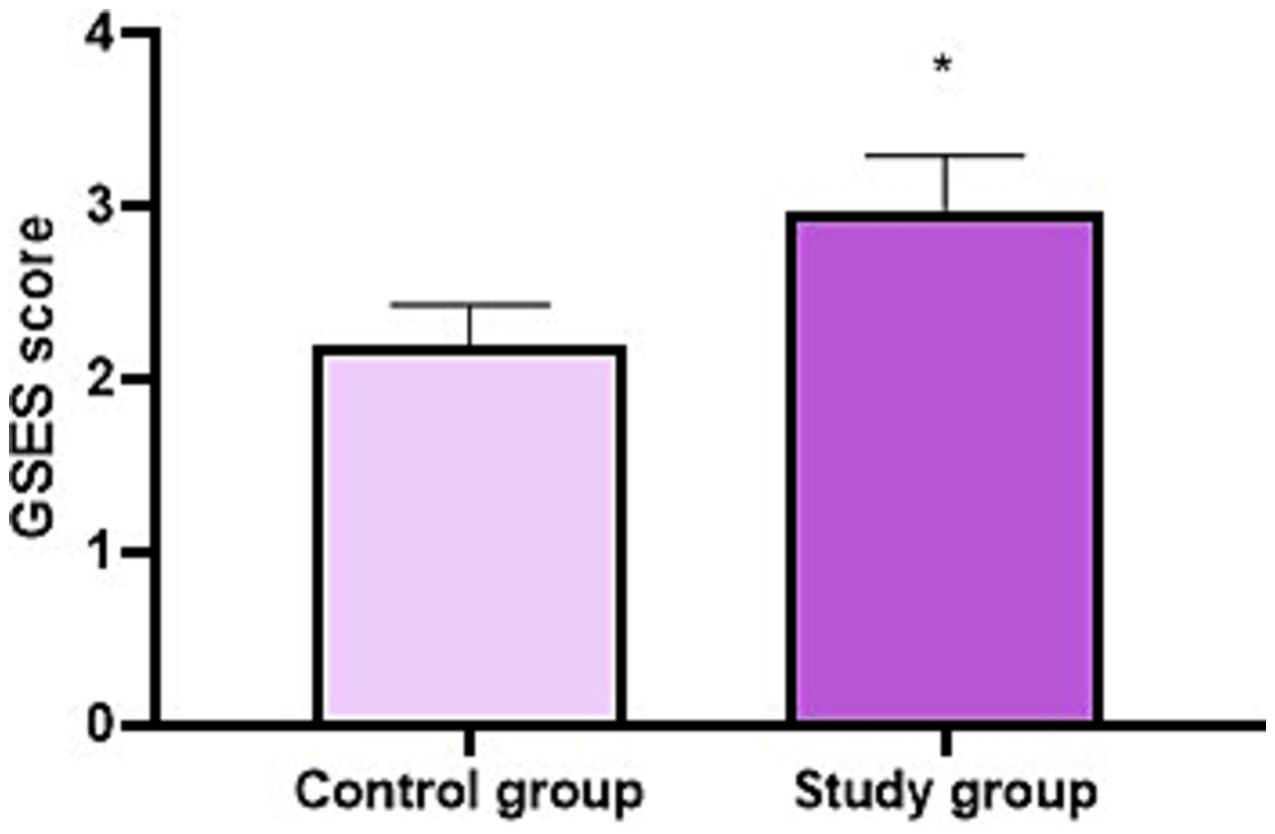
GSES score of the two groups. ^*^p < 0.05.

After 2 years of training, GSES score in the SG was higher compared to the CG (*p* < 0.05, Figure 3).

### Training satisfaction of nurses in both groups

After 2 years of training, the training satisfaction of nurses in the SG was higher compared to the CG (*p* < 0.05, Table 1).

**Table 1:** Training satisfaction of nurses in both groups (*n*, %).

Groups	Very satisfied	Satisfied	Unsatisfied	Satisfaction rate
Control group (*n* = 130)	70	40	20	110 (84.62%)
Study group (*n* = 130)	80	43	7	123 (94.62%)
*χ* ^2^	6.99			
*p*-value	<0.05			

### Postoperative wound healing conditions in two groups

**Figure 4:**
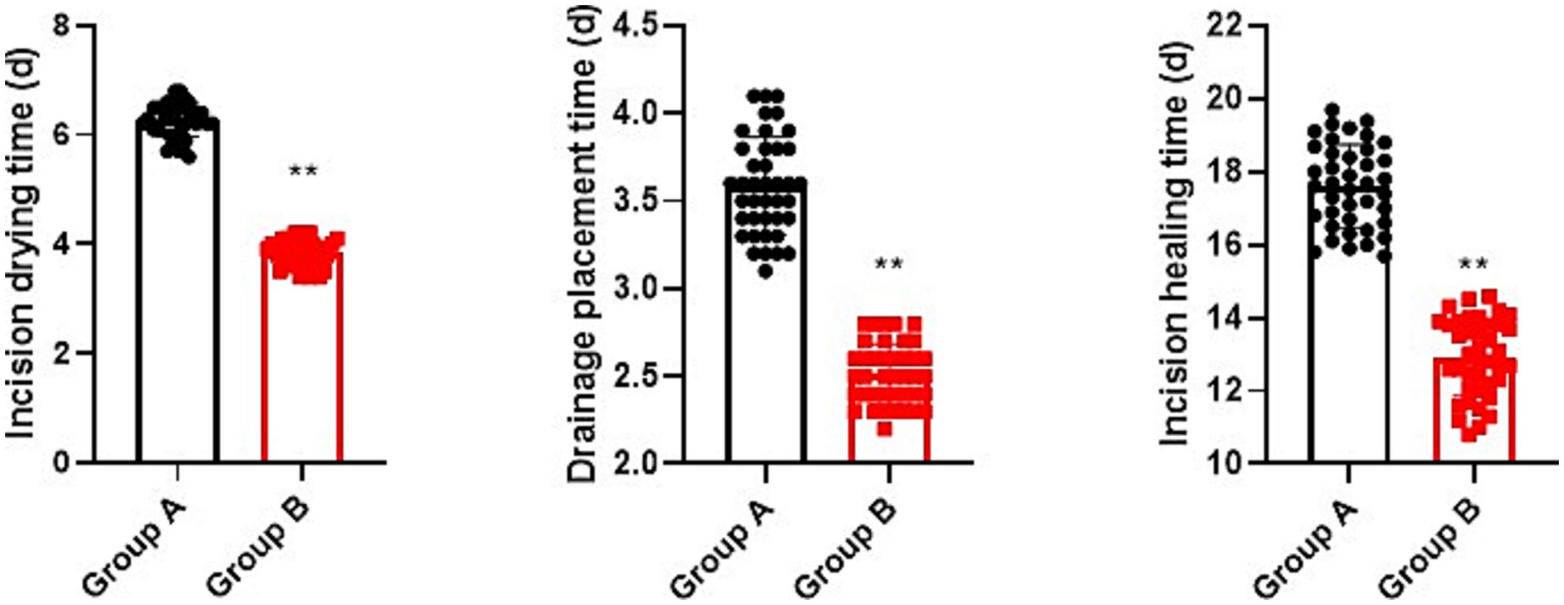
Postoperative wound healing conditions. ^**^p < 0.01.

Relative to group A, the incision drying time, drainage placement time, and incision healing time in group B were shorter (*p* < 0.05, Figure 4).

### Nutritional status in two groups

**Figure 5:**
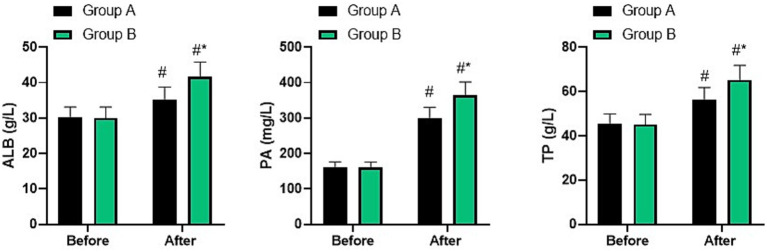
Nutritional status in two groups. ^#^*p* < 0.05, compared with before nursing, ^*^p < 0.05, compared with group A.

Before nursing, no difference was seen in ALB, PA, and TP levels between two groups (*p* > 0.05). After nursing, ALB, PA, and TP levels were elevated in two groups, and those in group B were higher than group A (*p* < 0.05, Figure 5).

### Immune function in two groups

**Figure 6:**
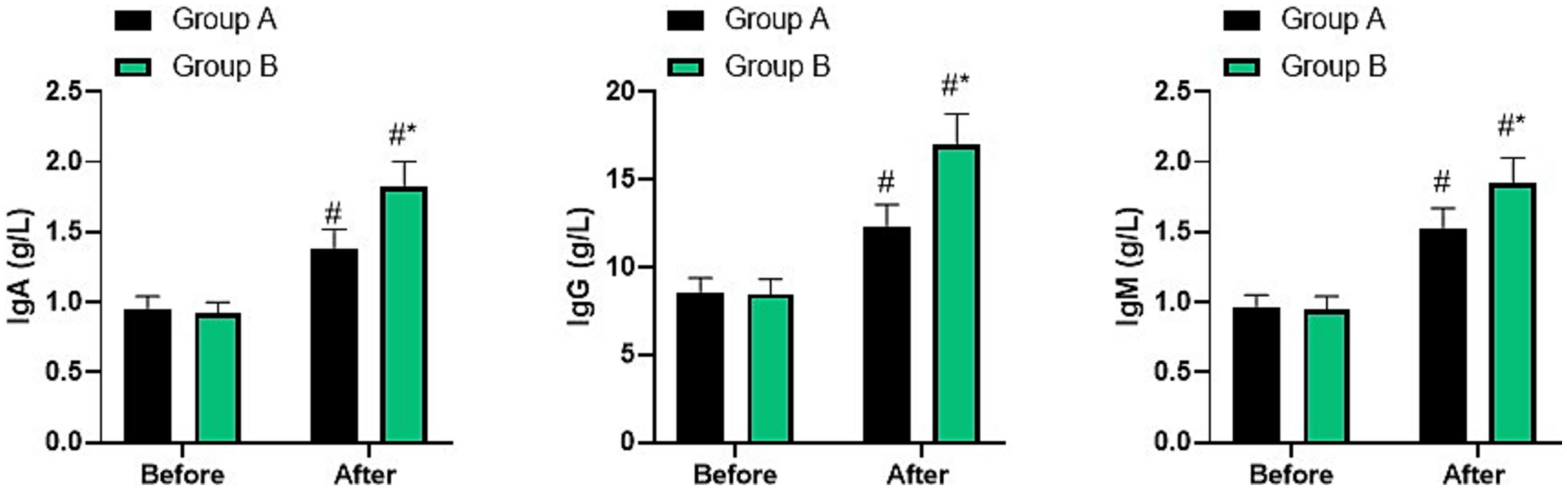
Immune function in two groups. ^#^*p* < 0.05, compared with before nursing, ^*^p < 0.05, compared with group A.

Before nursing, no difference was seen in IgA, IgG, and IgM levels between two groups (*p* > 0.05). After nursing, IgA, IgG, and IgM levels were elevated in two groups, and those in group B were higher than group A (*p* < 0.05, Figure 6).

### Occurrence of complications in two groups

Table 2 revealed that the occurrence of complications in group B was lower than group A (*p* < 0.05).

**Table 2:** Occurrence of complications in two groups.

Groups	Incision skin cracking	Incision skin infection	Incision skin necrosis	Total incidence rate
Group A (*n* = 40)	4	4	2	10 (25.00%)
Group B (*n* = 40)	1	1	1	3 (7.50%)
*χ* ^2^	4.501			
*p*-value	0.033			

## Discussion

This study aimed to assess the effect of the Six Sigma DMAIC model in quality management of new nurse training, as well as the impact of nutritional diet nursing on postoperative nutritional status in elderly patients with fractures.

The newly recruited nurses constitute a very important new force in the medical institution (16). Enhancing their comprehensive abilities through standardized training is crucial for improving the hospital’s nursing service level and promoting the implementation of high-quality nursing. This is of great value for the construction of an excellent nursing team in the hospital (17). During the initial stage of entering the hospital, new nurses are not yet proficient in their professional knowledge and need to be supplemented through training. The specific selection of management and training methods is of vital importance (18).

Six Sigma management is an implementation principle and technology that can strictly, centrally, and efficiently enhance the quality of enterprise process management (19). It encompasses many pioneering achievements in the field of management and aims to improve product and service quality, pursue “perfect quality” and “zero defects,” and significantly reduce quality costs (20). The total quality management method includes numerous measurement tools and methods, while Six Sigma extracts the essence and most effective methods of process management technology (21). DMAIC is the most important and classic improvement mode in Six Sigma management, mainly focusing on the quality improvement of existing processes (22). The Six Sigma DMAIC model follows a five-step cycle and can adopt various teaching methods to ensure that students can constantly consolidate and review during the learning process and fully apply the theoretical knowledge they have learned to practice, thereby enhancing their learning identification and satisfaction (23). This study applied it to the management and training of new nurses. Through this new mode, the management structure can be further optimized, and a clear training plan can be formulated to ensure targeted learning and training for nurses, thereby helping newly hired nurses adapt to the working environment and carry out work, and improve their comprehensive qualities.

The results of this study revealed that after 2 years of training, the theoretical and practical assessment scores, scores of each dimension of CCTDI 2000, GSES score, and training satisfaction of nurses in the SG were all higher than those of the CG. This suggested that applying the Six Sigma DMAIC model to the training and teaching of new nurses had a better effect, which could improve the teaching quality, assessment performance, and critical thinking ability of nurses, thereby enhancing their self-efficacy and recognition. The possible reasons may be as follows: the Six Sigma DMAIC model can optimize the management structure and formulate the initial training plan, thereby promoting the learning and training of new nurses, improving their adaptability, and enhancing their comprehensive qualities. At the same time, in the training process, the strength of the team should be fully utilized through brainstorming to promptly identify problems and jointly discuss and solve the deficiencies in the management training of new nurses. The head nurse and the responsible nurse should strengthen supervision and play a leading role. During the training and teaching period, they should not only set strict requirements but also encourage nurses to create a comfortable teaching atmosphere. The Six Sigma DMAIC management model has changed the long-term “instructive” teaching method of teachers, replacing it with definition, measurement, analysis, and participation of nurses, thereby greatly improving the teaching quality.

Nutrition forms the fundamental basis for human survival, transformation, and metabolism (24). However, due to digestive and absorption functions, the elderly often suffer from underlying diseases such as diabetes and hypertension, which lead to more dietary restrictions and poor nutrient absorption (25). Fracture patients usually have a high demand for nutrition after surgery. Factors such as surgical trauma and inflammation increase the patient’s nutrient consumption, further aggravating the patient’s nutritional dysfunction and affecting the prognosis. This nutritional dysfunction is more significant in elderly fracture patients (26). Currently, most clinical routine care focuses mainly on disease monitoring and accident prevention for the elderly fracture patients, and the nutritional advice given to patients is rather rough, often lacking specific and targeted considerations for the patients (27). In addition, patients and their families lack understanding of the relationship between nutritional status and disease prognosis, resulting in insufficient postoperative nutritional supply for patients and poor prognosis (28). Therefore, it is particularly important to pay attention to and intervene in patients’ nutritional status in nursing work, which is conducive to promoting the postoperative rehabilitation of elderly fracture patients.

Nutritional diet nursing is a specific nursing intervention tailored to the nutritional status of patients (29). Elderly patients with fractures need to supplement a variety of nutrients after surgery to alleviate their pain symptoms, accelerate the recovery process, and help them recover by assessing their nutritional status and providing targeted nutritional support (30). In addition, personalized diet plans will be formulated for the patients, and the effects of improving their nutritional status will be achieved through strict implementation and supervision, thereby accelerating the recovery process of the patients (31). In our study, the results indicated that relative to group A, the incision drying time, drainage placement time, and incision healing time in group B were shorter. After nursing, the levels of ALB, PA, and TP, as well as IgA, IgG, and IgM in both groups increased, and the indicators of group B were higher than those of group A. The incidence of complications in group B was lower than that in group A. All these results suggested that nutritional diet nursing could promote postoperative incision healing, enhance postoperative nutritional status, and immune function in elderly patients with fractures, which was in line with previous studies (32, 33). This is because the inflammatory response of the body after surgery in elderly fracture patients will increase protein consumption, and the decrease in serum protein levels will reduce humoral immune indicators and increase the risk of postoperative infection. Nutritional diet care can provide targeted supplementation of the nutrients needed by the patients to meet their nutritional needs, thereby enhancing immunity.

This study has some limitations. First, we did not conduct an in-depth exploration of the possible implementation obstacles that the Six Sigma DMAIC model might encounter during its application in the training of new nurses. The resistance of nurses or staff to changes is a common problem when implementing new methods in the medical field. During the process of introducing the Six Sigma DMAIC model for new nurse training, some senior nurses might have doubts about the data-driven and process-optimization concepts emphasized by the model, fearing that it would increase their workload or affect the training outcomes. The complexity of medical processes cannot be ignored either. The new nurse training involves multiple departments and links, and the work processes and standards in different departments vary, which makes the unified application and promotion of the Six Sigma DMAIC model challenging. The limitations in resources also restrict the application of this model. The scarcity of time and funds may prevent providing sufficient training and practical opportunities for new nurses, and the shortage of personnel with specialized Six Sigma training will also affect the accuracy and effectiveness of model implementation. Moreover, in the medical environment, the results are often influenced by multiple factors, making it difficult to precisely measure the individual contribution of the Six Sigma DMAIC model to the training effect. This also brings certain difficulties to the evaluation process. Although this study did not address these implementation obstacles, they are crucial for the long-term application and promotion of the Six Sigma DMAIC model in the training of new nurses. In future research, we will conduct in-depth investigations and analyses on these issues. For instance, through questionnaires and interviews, we will understand the reasons and extent of nurses’ resistance to the changes, and develop corresponding communication and training strategies to alleviate their resistance; we will thoroughly review and optimize the medical processes to ensure that the Six Sigma DMAIC model can be closely integrated with the actual work processes; we will reasonably plan resources, strive for more time and financial support, and strengthen the training of relevant personnel; we will explore more scientific and reasonable evaluation methods to accurately measure the application effect of the Six Sigma DMAIC model in the training of new nurses. We believe that through these efforts, we can further improve the application system of the Six Sigma DMAIC model in the training of new nurses and provide strong support for improving the quality of nursing and cultivating outstanding nursing talents. Second, this study focused on training the core elements of the professional field, which may, to some extent, neglect other key weaknesses of new nurses upon entering the workplace, such as communication skills, leadership abilities, organizational skills, stress management, and situational judgment. Multiple studies have clearly confirmed that these areas are the core where new nurses lack competence, and they have a crucial impact on the overall nursing performance and the quality of patient care. This study did not cover the training of these abilities, which might pose certain challenges for new nurses in their actual work. In future research, we will fully take these limitations into account and further expand the research content. On the one hand, we plan to incorporate training in communication skills, leadership skills, organizational skills, stress management, and situational judgment into the application system of the Six Sigma DMAIC model and design more comprehensive and systematic training programs. On the other hand, we will conduct long-term follow-up studies to evaluate the long-term impact of these comprehensive ability training on the career development of new nurses, the overall performance of the nursing team, and the quality of patient care. At the same time, we will actively draw on research results from other related fields, continuously optimize training methods and evaluation indicators, and provide a more scientific and effective basis for improving the comprehensive quality of new nurses and the quality of nursing services. Third, Six Sigma heavily relies on quantitative metrics, which may cause overemphasis on measurable outcomes while potentially neglecting qualitative factors such as nurse morale, patient satisfaction, and team dynamics that profoundly influence care quality. During the implementation of this study, although we were aware of the significance of these non-quantitative factors, due to the inherent characteristics of the Six Sigma method, they were not given sufficient attention and in-depth evaluation. This may lead to certain deviations in our comprehensive understanding of the training effect of new nurses and provides an improvement direction for our subsequent research and practice. In future research, we will attempt to incorporate the assessment of non-quantitative factors into the application framework of the Six Sigma DMAIC model. For instance, we will employ methods such as questionnaires, interviews, and focus groups to collect subjective evaluations from nurses, patients, and team members regarding nurse morale, patient satisfaction, and team collaboration. We will then combine these qualitative data with quantitative indicators for comprehensive analysis. In addition, we will explore ways to integrate more elements that enhance nurse morale, improve patient experience, and promote team collaboration during the training process, such as conducting team-building activities and strengthening humanistic care training. Through these efforts, we aim to establish a more comprehensive and refined new nurse training system for Six Sigma, providing stronger support for improving nursing quality and cultivating high-quality nursing talents. Fourth, due to the limitation of sample size in this study, we were unable to explore potential confounding factors that may affect the nutritional status and rehabilitation outcomes, such as the patients’ comorbid conditions, medication use, compliance, or initial dietary habits. These factors are particularly complex and crucial in the elderly patient population. In future studies, we will increase the sample size and employ more advanced statistical analysis methods, such as multivariate regression analysis and propensity score matching, to eliminate the interference of confounding factors and more accurately assess the independent impact of nutritional diet care on the postoperative nutritional status and rehabilitation outcomes of elderly fracture patients. Fifth, we used a self-designed questionnaire to collect feedback on the satisfaction of new nurses regarding the training, but this questionnaire may have the problem of insufficient validation. Although designing the questionnaire can be tailored to the specific requirements and characteristics of the research to obtain information closely related to the research objectives, due to the lack of extensive professional validation and reliability and validity tests, the accuracy and reliability of the measurement results may be affected to some extent. To address these issues, in future research, we will take a series of measures to improve the situation. We will conduct a comprehensive validity and reliability test on the independently designed questionnaire, using methods such as pre-survey, expert review, and factor analysis, to ensure that the questionnaire has high content validity and structural validity. We will introduce external standards as references, such as referring to recognized training satisfaction assessment tools or indicators in the industry, and comparing the results of the designed questionnaire with them to verify the validity and accuracy of the questionnaire. Furthermore, our study was a single-center study, which may lead to the training and improvement measures seem to have specific institutional characteristics. This limits their applicability for promotion in different systems or among different groups of nurses. In future research, we will conduct multi-center studies to verify the application effect of the Six Sigma DMAIC model in different institutions and among different groups of nursing staff, thereby providing more sufficient evidence for the promotion and application of this model. Future research can further increase the sample size, extend the research period, and employ more precise measurement tools to assess the concentration and other potential interfering factors, to more comprehensively and deeply verify our research results.

## Conclusion

The application of Six Sigma DMAIC management in the training and teaching of new nurses is good, which can improve the teaching quality, improve the assessment performance and evaluation thinking ability of nurses, and is worthy of clinical promotion and application. In addition, nutritional diet nursing could promote postoperative incision healing and enhance postoperative nutritional status and immune function in elderly patients with fractures.

## Data Availability

The original contributions presented in the study are included in the article/supplementary material, further inquiries can be directed to the corresponding author.
